# 4,4-Dimethyl-2-[3-nitro-2-phenyl-1-(phenyl­sulfan­yl)prop­yl]-4,5-dihydro-1,3-oxazole

**DOI:** 10.1107/S1600536812012512

**Published:** 2012-03-31

**Authors:** Ignez Caracelli, Julio Zukerman-Schpector, José A. F. P. Villar, Alfredo R. M. Oliveira, Edward R. T. Tiekink

**Affiliations:** a BioMat-Departamento de Física, Universidade Federal de São Carlos, C.P. 676, 13565-905, São Carlos, SP, Brazil; b Laboratório de Cristalografia, Estereodinâmica e, Modelagem Molecular, Universidade Federal de São Carlos, Departamento de Química, C.P. 676, 13565-905, São Carlos, SP, Brazil; c Universidade Federal de São João del Rei, Av. Sebastião Goncalves Coelho, 400, 35501-296, Divinópolis, MG, Brazil; d Universidade Federal do Paraná, Departamento de Química, C.P. 19081, 81531-990, Curitiba, PR, Brazil; e Department of Chemistry, University of Malaya, 50603 Kuala Lumpur, Malaysia

## Abstract

In the title compound, C_20_H_22_N_2_O_3_S, the oxazoline ring is planar (r.m.s. deviation = 0.045 Å) and forms dihedral angles of 47.24 (8) and 10.11 (8)° with the S- and C-bound phenyl rings, respectively. The nitro group lies to the same side of the mol­ecule as the oxazoline ring but is orientated so as not to inter­act with the ring. Linear supra­molecular chains along [010] are formed *via* C—H⋯O and C—H⋯S contacts. Chains are consolidated into a three-dimensional architecture by C—H⋯π and van der Waals inter­actions.

## Related literature

For background on the biological activities of Rolipram, see: de Visser *et al.* (2008 ▶). For the synthesis of the title compound, see Villar (2008 ▶); Oliveira *et al.* (2007 ▶).
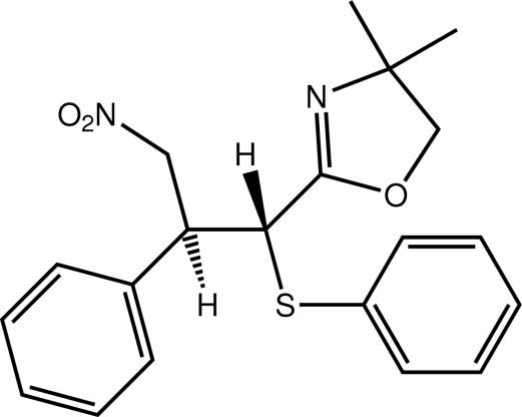


## Experimental

### 

#### Crystal data

C_20_H_22_N_2_O_3_S
*M* *_r_* = 370.47
Monoclinic, 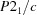

*a* = 15.339 (3) Å
*b* = 5.7040 (8) Å
*c* = 22.786 (4) Å
β = 107.166 (2)°
*V* = 1904.8 (6) Å^3^
*Z* = 4
Mo *K*α radiation
μ = 0.19 mm^−1^
*T* = 98 K
0.25 × 0.15 × 0.15 mm

#### Data collection

Rigaku AFC12/SATURN724 diffractometer
Absorption correction: multi-scan (*ABSCOR*; Higashi, 1995 ▶) *T*_min_ = 0.809, *T*_max_ = 1.000
15389 measured reflections
4362 independent reflections
4146 reflections with *I* > 2σ(*I*)
*R*_int_ = 0.035
Standard reflections: 0

#### Refinement

*R*[*F* ^2^ > 2σ(*F* ^2^)] = 0.047
*wR*(*F* ^2^) = 0.114
*S* = 1.11
4362 reflections
235 parameters
H-atom parameters constrained
Δρ_max_ = 0.28 e Å^−3^
Δρ_min_ = −0.33 e Å^−3^

### 

Data collection: *CrystalClear* (Molecular Structure Corporation & Rigaku, 2005 ▶); cell refinement: *CrystalClear*; data reduction: *CrystalClear*; program(s) used to solve structure: *SIR92* (Altomare *et al.*, 1999 ▶); program(s) used to refine structure: *SHELXL97* (Sheldrick, 2008 ▶); molecular graphics: *ORTEP-3* (Farrugia, 1997 ▶), *DIAMOND* (Brandenburg, 2006 ▶) and *MarvinSketch* (ChemAxon, 2009 ▶); software used to prepare material for publication: *publCIF* (Westrip, 2010 ▶).

## Supplementary Material

Crystal structure: contains datablock(s) global, I. DOI: 10.1107/S1600536812012512/qk2035sup1.cif

Structure factors: contains datablock(s) I. DOI: 10.1107/S1600536812012512/qk2035Isup2.hkl

Supplementary material file. DOI: 10.1107/S1600536812012512/qk2035Isup3.cml

Additional supplementary materials:  crystallographic information; 3D view; checkCIF report

## Figures and Tables

**Table 1:** Hydrogen-bond geometry (Å, °) *Cg*2 and *Cg*3 are the centroids of the C7–C12 and C15–C20 rings, respectively.

*D*—H⋯*A*	*D*—H	H⋯*A*	*D*⋯*A*	*D*—H⋯*A*
C14—H14*B*⋯O3^i^	0.99	2.52	3.376 (2)	145
C20—H20⋯S1^ii^	0.95	2.79	3.7194 (19)	166
C8—H8⋯*Cg*2^iii^	0.95	2.71	3.4345 (18)	134
C17—H17⋯*Cg*3^iv^	0.95	2.99	3.712 (2)	134

Symmetry codes: (i) 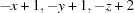; (ii) 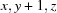; (iii) 
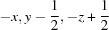
; (iv) 
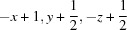
.

**Fig. 1.**
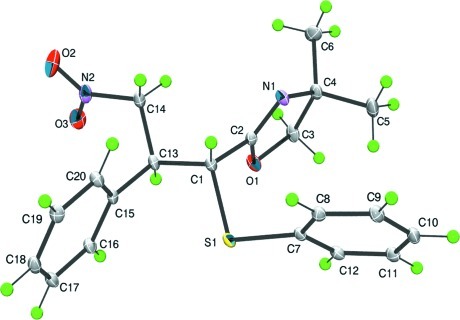
The molecular structure of compound (I) showing atom labelling scheme and displacement ellipsoids at the 50% probability level (arbitrary spheres for the H atoms).

**Fig. 2.**
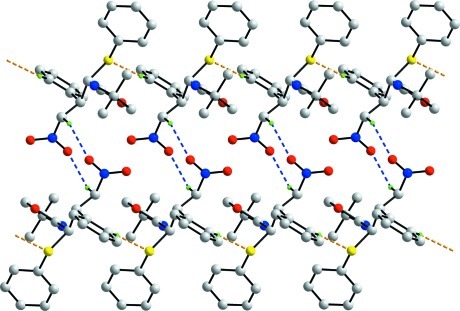
Supramolecular chain in (I) extending along [010] and sustained by C14—H14B···O3 and C20—H20···S1 contacts, shown as orange and blue dashed lines, respectively.

**Fig. 3.**
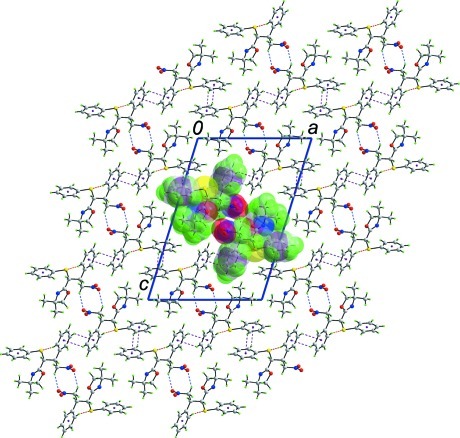
A view of the unit cell contents of (I) shown in projection down the b axis. One supramolecular chain is highlighted in space-filling mode. The C—H···O, C—H···S and C—H···π contacts are shown as orange, blue and purple dashed lines, respectively.

## supplementary crystallographic information

### Comment

While developing a new route aiming at the synthesis of 4-[3-(cyclopentyloxy)-4-methoxyphenyl]pyrrolidin-2-one (Rolipram), a phosphodiesterase-4 inhibitor which has been shown to have anti-inflammatory properties (de Visser *et al.*, 2008), the title compound, (I), was obtained during a systematic study of the addition reaction of oxazolines to nitroestyrene (Villar, 2008; Oliveira *et al.* 2007). The crystals were crystallographically characterized and the results are now reported herein.

In (I), Fig. 1, the oxazoline ring is planar (r.m.s. deviation = 0.045 Å) with the maximum deviations being 0.036 (2) Å for the C3 atom and -0.038 (2) Å for the C4 atom. The five-membered ring and the S-bound phenyl ring (C7–C12) are proximate and make a dihedral angle of 47.24 (8)°. The dihedral angles formed by these rings and the C-bound phenyl ring (C15–C20) are 10.11 (8) and 57.13 (8)°, respectively. The nitro group lies to the same side of the molecule as the oxazoline ring but is orientated away from the ring.

In the crystal packing, inversion related molecules are linked *via* C14—H14B···O3 contacts with the resultant dimeric aggregates connected into a linear supramolecular chain along [010] *via* C20—H20···S1 contacts, Fig. 2 and Table 1. Chains are consolidated in the three-dimensional packing by C—H···π and van der Waals interactions, Fig. 3 and Table 1.

### Experimental

The detailed synthesis of the title compound is described in a Ph.D. thesis (Villar, 2008). Crystals were grown by slow evaporation from an ethylacetate/ hexane solution held at 293 K. ^1^H-NMR (CDCl_3_, 400 MHz): δ (p.p.m.) 1.07 (*s*, 3H); 1.18 (*s*, 3H); 3.87(*d*, 1H, J = 8.15 Hz); 3.93 (*d*, 1H, J = 8.15 Hz); 3.98 (*ddd*, 1H, J = 8.43, 9.15, 5.27 Hz); 4.17 (*d*, 1H, J = 8.43 Hz); 4.97 (*dd*, 1H, J = 13.25, 5.27 Hz); 5.01 (*dd*, 1H, J = 13.25, 9.15 Hz); 7.18–7.34 (*m*, 8H); 7.38–7.42 (*m*, 2H); ^13^C (CDCl_3_, 100 MHz) δ (p.p.m.) 162.01; 136.34; 133.75; 132.43; 129.08; 128.91; 128.55; 128.39; 127.93; 79.44; 77.22; 67.44; 49.93; 45.85; 28.04. Analysis found: C 64.83, H 5.97, N 7.61, S 8.85%. C_20_H_22_N_2_O_3_S requires: C 64.84, H 5.99, N 7.56, S 8.65%.

### Refinement

The H atoms were geometrically placed (C—H = 0.95–1.00 Å) and refined as riding with *U_iso_*(H) = 1.2*U_eq_*(C) and *U_iso_*(H) = 1.5*U_eq_*(methyl-C).

### Crystal data

**Table:**

C_20_H_22_N_2_O_3_S	*F*(000) = 784
*M**_r_* = 370.47	*D*_x_ = 1.292 Mg m^−^^3^
Monoclinic, *P*2_1_/*c*	Mo *K*α radiation, λ = 0.71073 Å
Hall symbol: -P 2ybc	Cell parameters from 10219 reflections
*a* = 15.339 (3) Å	θ = 1.9–40.6°
*b* = 5.7040 (8) Å	µ = 0.19 mm^−^^1^
*c* = 22.786 (4) Å	*T* = 98 K
β = 107.166 (2)°	Block, colourless
*V* = 1904.8 (6) Å^3^	0.25 × 0.15 × 0.15 mm
*Z* = 4	

### Data collection

**Table:**

Rigaku AFC12K/SATURN724 diffractometer	4362 independent reflections
Radiation source: fine-focus sealed tube	4146 reflections with *I* > 2σ(*I*)
Graphite monochromator	*R*_int_ = 0.035
ω scans	θ_max_ = 27.5°, θ_min_ = 2.6°
Absorption correction: multi-scan (*ABSCOR*; Higashi, 1995)	*h* = −19→19
*T*_min_ = 0.809, *T*_max_ = 1.000	*k* = −7→7
15389 measured reflections	*l* = −29→29

### Refinement

**Table:**

Refinement on *F*^2^	Primary atom site location: structure-invariant direct methods
Least-squares matrix: full	Secondary atom site location: difference Fourier map
*R*[*F*^2^ > 2σ(*F*^2^)] = 0.047	Hydrogen site location: inferred from neighbouring sites
*wR*(*F*^2^) = 0.114	H-atom parameters constrained
*S* = 1.11	*w* = 1/[σ^2^(*F*_o_^2^) + (0.0504*P*)^2^ + 0.9546*P*] where *P* = (*F*_o_^2^ + 2*F*_c_^2^)/3
4362 reflections	(Δ/σ)_max_ < 0.001
235 parameters	Δρ_max_ = 0.28 e Å^−^^3^
0 restraints	Δρ_min_ = −0.33 e Å^−^^3^

### Special details

**Table:**

Geometry. All e.s.d.'s (except the e.s.d. in the dihedral angle between two l.s. planes) are estimated using the full covariance matrix. The cell e.s.d.'s are taken into account individually in the estimation of e.s.d.'s in distances, angles and torsion angles; correlations between e.s.d.'s in cell parameters are only used when they are defined by crystal symmetry. An approximate (isotropic) treatment of cell e.s.d.'s is used for estimating e.s.d.'s involving l.s. planes.
Refinement. Refinement of *F*^2^ against ALL reflections. The weighted *R*-factor *wR* and goodness of fit *S* are based on *F*^2^, conventional *R*-factors *R* are based on *F*, with *F* set to zero for negative *F*^2^. The threshold expression of *F*^2^ > 2σ(*F*^2^) is used only for calculating *R*-factors(gt) *etc*. and is not relevant to the choice of reflections for refinement. *R*-factors based on *F*^2^ are statistically about twice as large as those based on *F*, and *R*- factors based on ALL data will be even larger.

### Fractional atomic coordinates and isotropic or equivalent isotropic displacement parameters (Å^2^)

**Table:**

	*x*	*y*	*z*	*U*_iso_*/*U*_eq_	
S1	0.19877 (2)	0.17504 (7)	0.813988 (16)	0.02204 (11)	
O1	0.27948 (8)	0.1328 (2)	0.95576 (5)	0.0253 (2)	
O2	0.50324 (9)	0.8943 (3)	0.91166 (7)	0.0465 (4)	
O3	0.53747 (8)	0.5316 (2)	0.93550 (6)	0.0361 (3)	
N1	0.19037 (9)	0.4465 (2)	0.95984 (6)	0.0224 (3)	
N2	0.48312 (9)	0.6943 (3)	0.92173 (6)	0.0265 (3)	
C1	0.24888 (9)	0.4033 (3)	0.87082 (6)	0.0180 (3)	
H1	0.2159	0.5540	0.8571	0.022*	
C2	0.23715 (9)	0.3355 (3)	0.93164 (6)	0.0176 (3)	
C3	0.25828 (11)	0.1010 (3)	1.01329 (7)	0.0271 (3)	
H3A	0.3142	0.1120	1.0486	0.033*	
H3B	0.2293	−0.0534	1.0144	0.033*	
C4	0.19137 (10)	0.3029 (3)	1.01474 (7)	0.0230 (3)	
C5	0.09488 (12)	0.2111 (4)	1.00615 (8)	0.0344 (4)	
H5A	0.0754	0.1173	0.9685	0.052*	
H5B	0.0531	0.3436	1.0030	0.052*	
H5C	0.0943	0.1135	1.0414	0.052*	
C6	0.22443 (14)	0.4495 (3)	1.07286 (8)	0.0347 (4)	
H6A	0.2863	0.5063	1.0772	0.052*	
H6B	0.2248	0.3532	1.1086	0.052*	
H6C	0.1835	0.5835	1.0703	0.052*	
C7	0.09006 (10)	0.1354 (3)	0.82616 (6)	0.0190 (3)	
C8	0.02137 (10)	0.3010 (3)	0.80472 (7)	0.0205 (3)	
H8	0.0337	0.4424	0.7866	0.025*	
C9	−0.06542 (10)	0.2590 (3)	0.80984 (7)	0.0240 (3)	
H9	−0.1119	0.3737	0.7961	0.029*	
C10	−0.08439 (10)	0.0499 (3)	0.83492 (7)	0.0248 (3)	
H10	−0.1443	0.0195	0.8371	0.030*	
C11	−0.01562 (11)	−0.1148 (3)	0.85681 (7)	0.0250 (3)	
H11	−0.0285	−0.2575	0.8741	0.030*	
C12	0.07213 (11)	−0.0708 (3)	0.85336 (7)	0.0224 (3)	
H12	0.1196	−0.1810	0.8695	0.027*	
C13	0.35030 (9)	0.4353 (3)	0.87367 (6)	0.0184 (3)	
H13	0.3850	0.2917	0.8923	0.022*	
C14	0.38517 (10)	0.6438 (3)	0.91664 (7)	0.0220 (3)	
H14A	0.3476	0.7838	0.9006	0.026*	
H14B	0.3794	0.6082	0.9579	0.026*	
C15	0.36148 (9)	0.4727 (3)	0.81003 (6)	0.0179 (3)	
C16	0.40566 (10)	0.3047 (3)	0.78483 (7)	0.0224 (3)	
H16	0.4303	0.1688	0.8079	0.027*	
C17	0.41408 (11)	0.3347 (3)	0.72591 (7)	0.0255 (3)	
H17	0.4443	0.2190	0.7090	0.031*	
C18	0.37849 (10)	0.5328 (3)	0.69199 (7)	0.0251 (3)	
H18	0.3839	0.5528	0.6518	0.030*	
C19	0.33489 (11)	0.7019 (3)	0.71705 (7)	0.0245 (3)	
H19	0.3108	0.8384	0.6941	0.029*	
C20	0.32642 (10)	0.6719 (3)	0.77579 (7)	0.0220 (3)	
H20	0.2965	0.7883	0.7927	0.026*	

### Atomic displacement parameters (Å^2^)

**Table:**

	*U*^11^	*U*^22^	*U*^33^	*U*^12^	*U*^13^	*U*^23^
S1	0.01822 (19)	0.0306 (2)	0.01930 (19)	0.00031 (14)	0.00869 (14)	−0.00508 (14)
O1	0.0314 (6)	0.0267 (6)	0.0215 (5)	0.0077 (5)	0.0136 (5)	0.0083 (4)
O2	0.0322 (7)	0.0477 (8)	0.0588 (9)	−0.0099 (6)	0.0124 (6)	0.0209 (7)
O3	0.0209 (6)	0.0469 (8)	0.0387 (7)	0.0037 (5)	0.0060 (5)	0.0024 (6)
N1	0.0230 (6)	0.0289 (7)	0.0175 (6)	0.0028 (5)	0.0094 (5)	0.0014 (5)
N2	0.0193 (6)	0.0395 (8)	0.0200 (6)	−0.0034 (5)	0.0048 (5)	0.0044 (6)
C1	0.0162 (6)	0.0228 (7)	0.0163 (6)	0.0023 (5)	0.0066 (5)	0.0009 (5)
C2	0.0151 (6)	0.0206 (7)	0.0169 (6)	0.0001 (5)	0.0047 (5)	0.0014 (5)
C3	0.0301 (8)	0.0326 (9)	0.0223 (7)	0.0010 (7)	0.0135 (6)	0.0094 (7)
C4	0.0239 (7)	0.0304 (8)	0.0167 (7)	−0.0033 (6)	0.0089 (6)	0.0007 (6)
C5	0.0269 (8)	0.0541 (11)	0.0263 (8)	−0.0091 (8)	0.0143 (7)	−0.0035 (8)
C6	0.0479 (11)	0.0374 (10)	0.0208 (8)	−0.0101 (8)	0.0133 (7)	−0.0045 (7)
C7	0.0185 (6)	0.0234 (7)	0.0167 (6)	0.0006 (5)	0.0077 (5)	−0.0029 (5)
C8	0.0215 (7)	0.0229 (7)	0.0173 (6)	−0.0005 (5)	0.0058 (5)	−0.0001 (5)
C9	0.0198 (7)	0.0304 (8)	0.0216 (7)	0.0043 (6)	0.0059 (6)	0.0008 (6)
C10	0.0216 (7)	0.0329 (8)	0.0228 (7)	−0.0028 (6)	0.0110 (6)	−0.0037 (6)
C11	0.0319 (8)	0.0240 (8)	0.0232 (7)	−0.0022 (6)	0.0142 (6)	−0.0007 (6)
C12	0.0261 (7)	0.0230 (7)	0.0200 (7)	0.0048 (6)	0.0099 (6)	−0.0001 (6)
C13	0.0156 (6)	0.0238 (7)	0.0175 (6)	0.0032 (5)	0.0074 (5)	0.0034 (5)
C14	0.0177 (7)	0.0307 (8)	0.0190 (7)	−0.0012 (6)	0.0076 (5)	0.0011 (6)
C15	0.0149 (6)	0.0226 (7)	0.0180 (6)	−0.0001 (5)	0.0073 (5)	0.0022 (5)
C16	0.0211 (7)	0.0238 (7)	0.0250 (7)	0.0026 (6)	0.0108 (6)	0.0015 (6)
C17	0.0245 (7)	0.0311 (8)	0.0247 (8)	−0.0004 (6)	0.0133 (6)	−0.0044 (6)
C18	0.0221 (7)	0.0373 (9)	0.0168 (6)	−0.0059 (6)	0.0071 (6)	−0.0012 (6)
C19	0.0238 (7)	0.0295 (8)	0.0193 (7)	0.0002 (6)	0.0051 (6)	0.0059 (6)
C20	0.0229 (7)	0.0247 (8)	0.0199 (7)	0.0038 (6)	0.0086 (6)	0.0023 (6)

### Geometric parameters (Å, º)

**Table:**

S1—C7	1.7837 (15)	C8—C9	1.391 (2)
S1—C1	1.8372 (15)	C8—H8	0.9500
O1—C2	1.3601 (18)	C9—C10	1.390 (2)
O1—C3	1.4520 (17)	C9—H9	0.9500
O2—N2	1.221 (2)	C10—C11	1.390 (2)
O3—N2	1.2250 (19)	C10—H10	0.9500
N1—C2	1.2652 (19)	C11—C12	1.394 (2)
N1—C4	1.4914 (19)	C11—H11	0.9500
N2—C14	1.5004 (19)	C12—H12	0.9500
C1—C2	1.4996 (19)	C13—C15	1.5246 (18)
C1—C13	1.5487 (19)	C13—C14	1.533 (2)
C1—H1	1.0000	C13—H13	1.0000
C3—C4	1.549 (2)	C14—H14A	0.9900
C3—H3A	0.9900	C14—H14B	0.9900
C3—H3B	0.9900	C15—C16	1.392 (2)
C4—C6	1.521 (2)	C15—C20	1.393 (2)
C4—C5	1.527 (2)	C16—C17	1.397 (2)
C5—H5A	0.9800	C16—H16	0.9500
C5—H5B	0.9800	C17—C18	1.387 (2)
C5—H5C	0.9800	C17—H17	0.9500
C6—H6A	0.9800	C18—C19	1.389 (2)
C6—H6B	0.9800	C18—H18	0.9500
C6—H6C	0.9800	C19—C20	1.393 (2)
C7—C8	1.392 (2)	C19—H19	0.9500
C7—C12	1.394 (2)	C20—H20	0.9500
			
C7—S1—C1	101.31 (6)	C7—C8—H8	120.1
C2—O1—C3	105.29 (11)	C10—C9—C8	120.25 (14)
C2—N1—C4	106.52 (13)	C10—C9—H9	119.9
O2—N2—O3	124.43 (14)	C8—C9—H9	119.9
O2—N2—C14	117.89 (14)	C9—C10—C11	119.91 (14)
O3—N2—C14	117.67 (14)	C9—C10—H10	120.0
C2—C1—C13	112.63 (11)	C11—C10—H10	120.0
C2—C1—S1	109.23 (10)	C10—C11—C12	120.06 (15)
C13—C1—S1	108.62 (9)	C10—C11—H11	120.0
C2—C1—H1	108.8	C12—C11—H11	120.0
C13—C1—H1	108.8	C7—C12—C11	119.83 (14)
S1—C1—H1	108.8	C7—C12—H12	120.1
N1—C2—O1	119.64 (13)	C11—C12—H12	120.1
N1—C2—C1	125.53 (14)	C15—C13—C14	112.52 (12)
O1—C2—C1	114.79 (12)	C15—C13—C1	111.73 (11)
O1—C3—C4	104.63 (12)	C14—C13—C1	105.96 (11)
O1—C3—H3A	110.8	C15—C13—H13	108.8
C4—C3—H3A	110.8	C14—C13—H13	108.8
O1—C3—H3B	110.8	C1—C13—H13	108.8
C4—C3—H3B	110.8	N2—C14—C13	110.55 (12)
H3A—C3—H3B	108.9	N2—C14—H14A	109.5
N1—C4—C6	110.28 (13)	C13—C14—H14A	109.5
N1—C4—C5	108.13 (13)	N2—C14—H14B	109.5
C6—C4—C5	111.19 (13)	C13—C14—H14B	109.5
N1—C4—C3	103.49 (11)	H14A—C14—H14B	108.1
C6—C4—C3	111.99 (14)	C16—C15—C20	119.01 (13)
C5—C4—C3	111.44 (14)	C16—C15—C13	120.08 (13)
C4—C5—H5A	109.5	C20—C15—C13	120.90 (13)
C4—C5—H5B	109.5	C15—C16—C17	120.45 (14)
H5A—C5—H5B	109.5	C15—C16—H16	119.8
C4—C5—H5C	109.5	C17—C16—H16	119.8
H5A—C5—H5C	109.5	C18—C17—C16	120.16 (15)
H5B—C5—H5C	109.5	C18—C17—H17	119.9
C4—C6—H6A	109.5	C16—C17—H17	119.9
C4—C6—H6B	109.5	C17—C18—C19	119.66 (14)
H6A—C6—H6B	109.5	C17—C18—H18	120.2
C4—C6—H6C	109.5	C19—C18—H18	120.2
H6A—C6—H6C	109.5	C18—C19—C20	120.17 (15)
H6B—C6—H6C	109.5	C18—C19—H19	119.9
C8—C7—C12	120.02 (13)	C20—C19—H19	119.9
C8—C7—S1	120.32 (11)	C19—C20—C15	120.54 (14)
C12—C7—S1	119.50 (11)	C19—C20—H20	119.7
C9—C8—C7	119.85 (14)	C15—C20—H20	119.7
C9—C8—H8	120.1		
			
C7—S1—C1—C2	48.49 (11)	C8—C7—C12—C11	2.6 (2)
C7—S1—C1—C13	171.69 (10)	S1—C7—C12—C11	−172.84 (12)
C4—N1—C2—O1	−3.18 (18)	C10—C11—C12—C7	−2.0 (2)
C4—N1—C2—C1	174.70 (13)	C2—C1—C13—C15	172.60 (12)
C3—O1—C2—N1	−1.22 (18)	S1—C1—C13—C15	51.47 (14)
C3—O1—C2—C1	−179.33 (12)	C2—C1—C13—C14	−64.52 (15)
C13—C1—C2—N1	123.03 (16)	S1—C1—C13—C14	174.35 (9)
S1—C1—C2—N1	−116.19 (15)	O2—N2—C14—C13	127.19 (16)
C13—C1—C2—O1	−58.99 (16)	O3—N2—C14—C13	−52.53 (18)
S1—C1—C2—O1	61.79 (14)	C15—C13—C14—N2	−56.37 (16)
C2—O1—C3—C4	4.81 (16)	C1—C13—C14—N2	−178.76 (11)
C2—N1—C4—C6	125.75 (15)	C14—C13—C15—C16	127.36 (14)
C2—N1—C4—C5	−112.49 (15)	C1—C13—C15—C16	−113.57 (15)
C2—N1—C4—C3	5.81 (16)	C14—C13—C15—C20	−53.86 (18)
O1—C3—C4—N1	−6.40 (16)	C1—C13—C15—C20	65.21 (18)
O1—C3—C4—C6	−125.16 (14)	C20—C15—C16—C17	−0.5 (2)
O1—C3—C4—C5	109.57 (14)	C13—C15—C16—C17	178.30 (14)
C1—S1—C7—C8	75.16 (13)	C15—C16—C17—C18	0.1 (2)
C1—S1—C7—C12	−109.45 (12)	C16—C17—C18—C19	0.4 (2)
C12—C7—C8—C9	−0.8 (2)	C17—C18—C19—C20	−0.4 (2)
S1—C7—C8—C9	174.58 (11)	C18—C19—C20—C15	0.0 (2)
C7—C8—C9—C10	−1.6 (2)	C16—C15—C20—C19	0.4 (2)
C8—C9—C10—C11	2.1 (2)	C13—C15—C20—C19	−178.36 (14)
C9—C10—C11—C12	−0.3 (2)		

### Hydrogen-bond geometry (Å, º)

Please define Cg2 and Cg3

**Table:**

*D*—H···*A*	*D*—H	H···*A*	*D*···*A*	*D*—H···*A*
C14—H14*B*···O3^i^	0.99	2.52	3.376 (2)	145
C20—H20···S1^ii^	0.95	2.79	3.7194 (19)	166
C8—H8···*Cg*2^iii^	0.95	2.71	3.4345 (18)	134
C17—H17···*Cg*3^iv^	0.95	2.99	3.712 (2)	134

Symmetry codes: (i) −*x*+1, −*y*+1, −*z*+2; (ii) *x*, *y*+1, *z*; (iii) −*x*, *y*−1/2, −*z*+1/2; (iv) −*x*+1, *y*+1/2, −*z*+1/2.
